# Use of headspace–gas chromatography–ion mobility spectrometry to detect volatile fingerprints of palm fibre oil and sludge palm oil in samples of crude palm oil

**DOI:** 10.1186/s13104-019-4263-7

**Published:** 2019-04-16

**Authors:** Abrizah Othman, Kirstie A. Goggin, Noor Idayu Tahir, Emma Brodrick, Rajinder Singh, Ravigadevi Sambanthamurthi, Ghulam K. A. Parveez, Antony N. Davies, Abdul J. Murad, Nor H. Muhammad, Umi S. Ramli, Denis J. Murphy

**Affiliations:** 1 Faculty of Computing, Engineering and Science, University of South Wales, Pontypridd, CF37 1DL UK; 2 Malaysian Palm Oil Board, No 6, Persiaran Institusi, Bandar Baru Bangi, 43000 Kajang, Selangor Malaysia; 3 IMSPEX Diagnostics Ltd., Ty Menter Navigation Park, Abercynon, CF45 4SN UK; 4 Nouryon b.V., Deventer, The Netherlands

**Keywords:** Gas chromatography–ion mobility spectrometry, Crude palm oil, Adulteration, Fingerprinting, Rapid analysis, Volatile organic compounds

## Abstract

**Objective:**

The addition of residual oils such as palm fibre oil (PFO) and sludge palm oil (SPO) to crude palm oil (CPO) can be problematic within supply chains. PFO is thought to aggravate the accumulation of monochloropropanediols (MCPDs) in CPO, whilst SPO is an acidic by-product of CPO milling and is not fit for human consumption. Traditional targeted techniques to detect such additives are costly, time-consuming and require highly trained operators. Therefore, we seek to assess the use of gas chromatography–ion mobility spectrometry (GC–IMS) for rapid, cost-effective screening of CPO for the presence of characteristic PFO and SPO volatile organic compound (VOC) fingerprints.

**Results:**

Lab-pressed CPO and commercial dispatch tank (DT) CPO were spiked with PFO and SPO, respectively. Both additives were detectable at concentrations of 1% and 10% (w/w) in spiked lab-pressed CPO, via seven PFO-associated VOCs and 21 SPO-associated VOCs. DT controls could not be distinguished from PFO-spiked DT CPO, suggesting these samples may have already contained low levels of PFO. DT controls were free of SPO. SPO was detected in all SPO-spiked dispatch tank samples by all 21 of the previously distinguished VOCs and had a significant fingerprint consisting of four spectral regions.

**Electronic supplementary material:**

The online version of this article (10.1186/s13104-019-4263-7) contains supplementary material, which is available to authorized users.

## Introduction

Palm oil is the most widely consumed vegetable oil in the world and is a key ingredient in many sectors of both the food and oleochemical industries [1]. The Malaysian palm oil industry is highly regulated [1, 2] and internationally traded palm oil must adhere to stringent quality standards [3], which among other stipulations specify zero adulteration. However, the important commercial value of palm oil also lends itself to the risk of adulteration with certain residual oils within international supply chains.

PFO and SPO are residual oils which are sometimes added to CPO at the mill or along supply chains, usually to increase oil volume [4, 5]. PFO is a vitamin rich oil which is extracted from pressed-palm fruits, usually via solvent extraction. It is sometimes added to improve oil extraction rates, although this practice is not recommended as it may aggravate the accumulation of monochloropropane-1,2-diols (MCPDs), which are carcinogens [6], in CPO [7]. SPO is a highly acidic by-product of the palm oil industry and is not fit for human consumption. Again, it is sometimes added at various points in supply chains, for example, where CPO has been illegally siphoned off, in order to boost oil volumes [8]. The addition of SPO to CPO destined for use in food can jeopardise quality [9], and with that consumer rights.

As food fraud can be a difficult area of crime to police, it is important that robust and cost-effective analytical methods are developed to detect such incidents [10]. Targeted analytical techniques such as gas chromatography–mass spectrometry (GC–MS) are most commonly employed for detection of adulteration; however, such techniques are costly, require highly trained personnel and complex sample preparation, plus a clean laboratory environment. GC–IMS is an emerging technique which detects VOCs present in sample headspace down to parts per billion (ppb) or even parts per trillion (ppt). GC–IMS is simple to use, requires minimal or no sample preparation, operates at atmospheric pressure and does not require an ultra-high vacuum inside the detector, satisfying the majority of criteria which industry now stipulates [11].

In this study we assess the use of GC–IMS for rapid screening analysis of CPO, specifically for detecting the presence of PFO and SPO via characteristic volatile fingerprints. Several techniques have previously been used by other researchers for analysing VOC content of CPO. These include proton transfer reaction-mass spectrometry (PTR-MS), gas chromatography–mass spectrometry and headspace solid-phase microextraction (HS-SPME) [12–14]. The first used a fingerprinting approach for discerning palm oil by geographical origin, the second intended to identify specific VOCs which contributed to palm oil flavour, whilst the latter was optimised to detect furfural content in CPO.

## Main text

### Methods

#### Samples

Pure hexane-extracted PFO and pure SPO were provided to determine characteristic VOC markers, whilst lab-pressed CPO (free of residual oils) was provided to enable a comparative spiking experiment. To determine whether distinguished PFO and SPO markers could be used in real mill conditions, 40 CPO samples were obtained from four geographically diverse mills (Additional file 1: Table S1). All CPO samples were manually spiked by weight. Prior to analysis, samples were melted in an agitated water bath for 15 min at 50 °C and 275 rpm to enable aliquots to be taken. 1 g of CPO sample was weighed into a 20 mL glass headspace vial. Due to high VOC concentration, headspace dilution of pure SPO was conducted, whereby 500 μL of pure SPO headspace was taken and injected into an empty 20 mL glass headspace vial. Each vial was closed with a polytetrafluorethylene (PTFE) septa-sealed magnetic screw cap. Samples were stored at 4 °C until analysis.

#### Reagents

All reagents used in this work were of reagent grade. 2-Butanone, 2-hexanone and 2-nonanone were supplied by Sigma-Aldrich (St. Louis, MO, USA). Working solutions of concentration 500 μg L^−1^ were prepared by diluting each stock solution (1 g L^−1^) with deionised water. Each solution was analysed to determine inter- and intraday variations of drift times, retention times and peak intensities. Nitrogen gas was generated by a Leman Instruments Nitrogen generator (Archamps, France).

#### Instrumentation and software

Analyses of CPO samples were performed on a commercially available GC-IMS instrument (FlavourSpec©) from Gesellschaft für Analytische Sensorsysteme mbH (G.A.S., Dortmund, Germany). Instrumentation and operational parameters are displayed in Additional file 2: Table S2. IMS data were acquired in positive mode and data analysis was conducted using Laboratory Analytical Viewer (LAV) software (v.2.0.0) from G.A.S.

#### Data treatment

Visual comparisons of spectra were conducted using LAV_plugin_Reporter (v1.2.12). Peaks of interest were then manually selected, and gallery plots were generated using LAV_Gallery_plugin, to better visualise them. The reduced ion mobility was calculated for each marker of interest using the following equation:

$$K_{0} = \frac{l}{tE} \left( {\frac{{T_{o} }}{T}} \right)\left( {\frac{P}{{P_{o} }}} \right)$$

where *l* is tube length (cm), *t* is drift time (s), *E* is electric field (V cm^−1^), *T* is drift tube temperature (K) and *P* is the combination of drift tube and operational pressure (kPa). Hill et al. [15] stated, ‘inherent in the definition of reduced mobility is the idea that the ratio of drift time for any two ions is independent of temperature and pressure’, comparisons are therefore facilitated by reporting as 1/*K*_0_.

### Results and discussion

#### Optimisation of GC–IMS parameters

An optimisation protocol similar to that used by Arroyo-Manzanares et al. [16] was followed. Several experimental variables of the FlavourSpec were assessed to develop an optimised method, which sought to obtain maximum information about the samples in the minimum possible time. These included sample weight (0.5–2 g), incubation time (2–30 min), incubation temperature (40–80 °C), column temperature (40–80 °C) and carrier gas (N_2_ 6.0) flow rate (2–150 mL min^−1^). The optimised parameters are displayed in Additional file 2: Table S2 and were selected to facilitate fast run time, optimal signal intensity and peak separation.

#### Quality control of GC–IMS method

2-Butanone, 2-hexanone and 2-nonanone were selected to measure repeatability and intermediate precision as they have been identified previously in palm oil samples [13, 17], and showed very different drift and retention times when studied using GC–IMS. Intraday variation was determined by measuring the working solutions repeatedly throughout the day, whilst interday precision was determined by analysing each working solution once a day, over the period of a week. The results are displayed in Additional file 3: Table S3.

#### Topographic plots of reference samples

Visual inspection of the spectra of three types of pure oils (lab-pressed CPO, PFO and SPO) was conducted to identify potential VOC markers for differentiating between them. The three topographic plots obtained for (a) lab-pressed CPO, (b) PFO and (c) SPO are shown in Additional file 4: Figure S1. With regards to GC-IMS spectra, the x-axis represents IMS drift time (ms), y-axis represents retention time (s) and z-axis represents peak intensity (V). There were many obvious differences in VOC composition meaning theoretically, detection of spiking by both residual oils could be possible.

To test this theory, lab-pressed CPO was deliberately spiked with PFO and SPO at 1% and 10% (w/w), respectively then compared to the spectra of un-spiked lab-pressed CPO. Sixteen signals were unique to PFO but only seven of these were detectable in lab-pressed CPO spiked with PFO (Additional file 5: Table S4). Four were detectable at a 1% level (w/w), whilst the remaining three were only detectable at a 10% level (w/w).

**Fig. 1:**
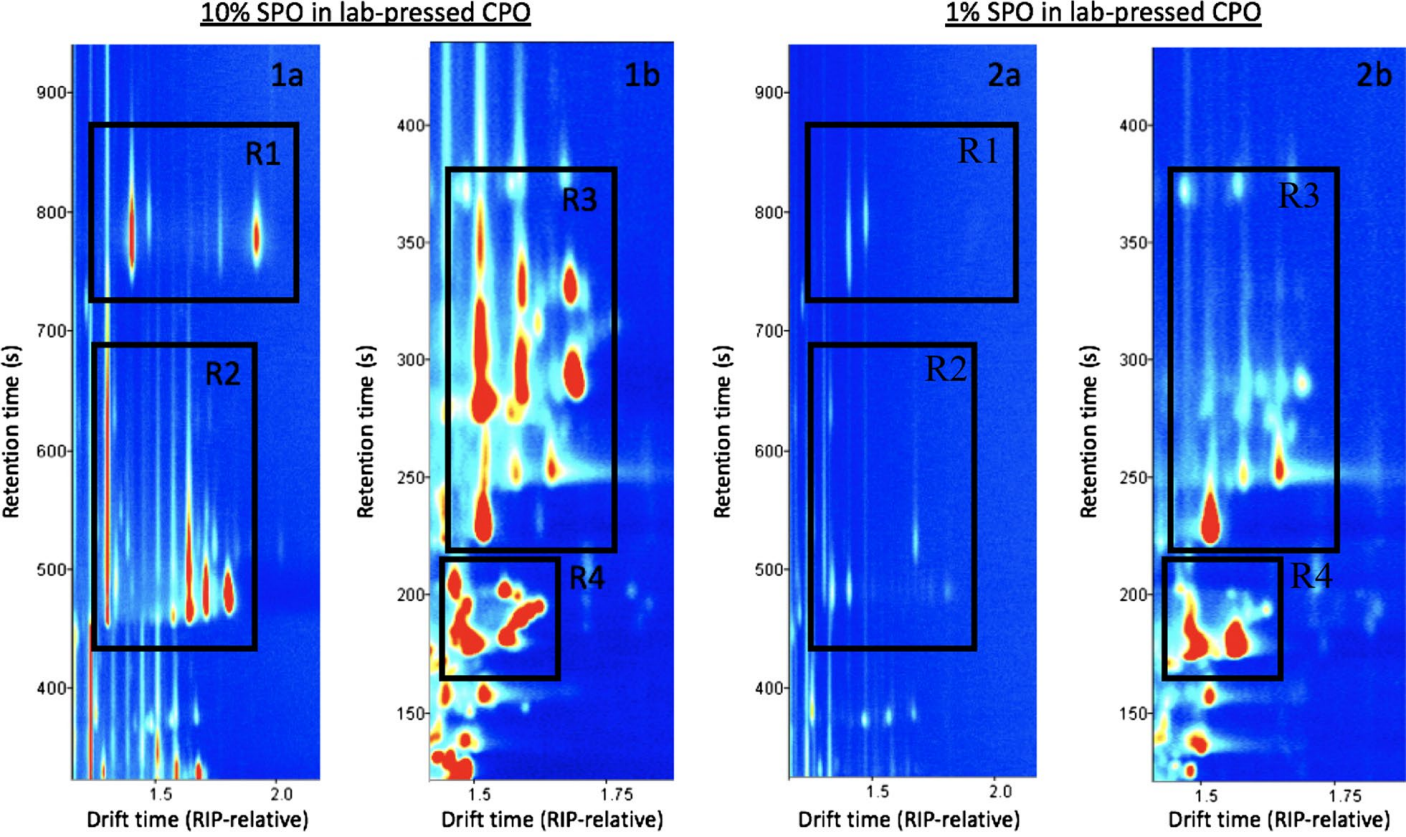
A reporter view showing the proposed SPO markers. 1a shows region 1 and 2 in 10% SPO + lab-pressed CPO, whilst 1b shows regions 3 and 4. This format is replicated for 1% SPO + lab-pressed CPO in panels 2a and 2b

SPO had many unique markers which spanned four main spectral regions, 21 of which were detectable in lab-pressed CPO spiked with SPO. The markers were present at 1% and 10% (w/w) spiking levels but differed slightly depending on spiking concentration (Fig. 1).

The system was able to detect as low as 1% SPO spiked in samples via detection of several lighter, less intense marker signals. When concentration was increased to 10% SPO, the system became saturated, meaning detection of the smaller peaks was not possible, instead detection of SPO was possible by several large and intense marker signals. The potential markers forming the SPO fingerprint are listed in Additional file 6: Table S5 and displayed in Additional file 7: Figure S2.

#### Topographical plots of spiked dispatch tank palm oil samples

To determine whether the two residual oils could be detected in real-life mill scenarios, CPO samples were obtained from dispatch tanks (DTs) at four geographically diverse processing mills and were spiked in the same manner as the lab-pressed CPO, using 4–5 different spiking concentrations (see Additional file 1: Table S1). They were analysed and compared to the spectra of the spiked lab-pressed CPO to determine whether the fingerprints matched.

Whilst lab-pressed CPO and control DT samples shared many common VOCs, control DT samples had many additional VOCs, which may be attributed to differences in storage conditions, bulking of oils from multiple plantations, etc.

**Fig. 2:**
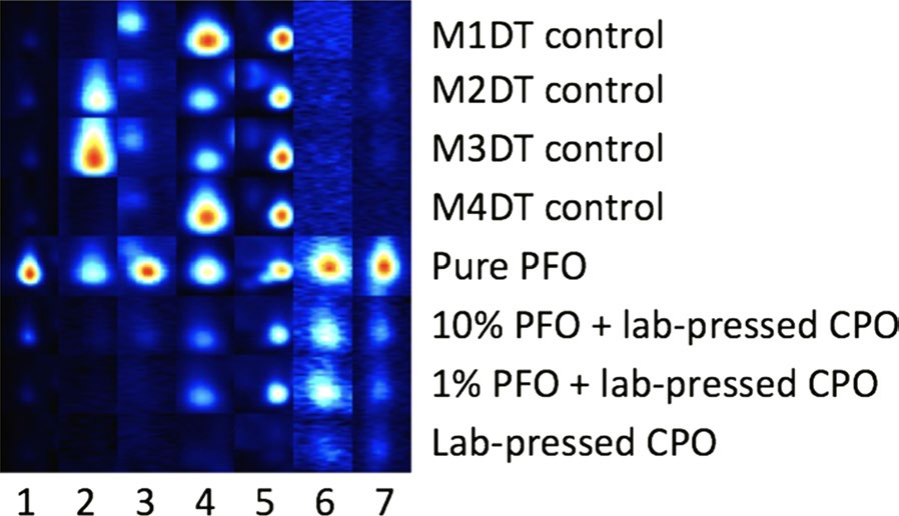
Gallery plot of seven suggested PFO markers in all of the DT control samples. Peaks 4, 5, 6 and 7 were detected in 1% PFO + lab-pressed CPO, whilst all seven were detected in 10% PFO + lab-pressed CPO

DT control samples were compared to the PFO-spiked DT samples for each mill location but there appeared to be no visible differences, suggesting that DT control samples may have already contained PFO. When compared to the profile of pure PFO, DT control samples contained several peaks that we determined as possible PFO markers, having been absent in lab-pressed CPO; however, the markers varied according to mill location (Fig. 2). Whilst seven PFO markers were successfully distinguished, we suggest this does not form a reliable fingerprint due to variability in mill samples.

**Fig. 3:**
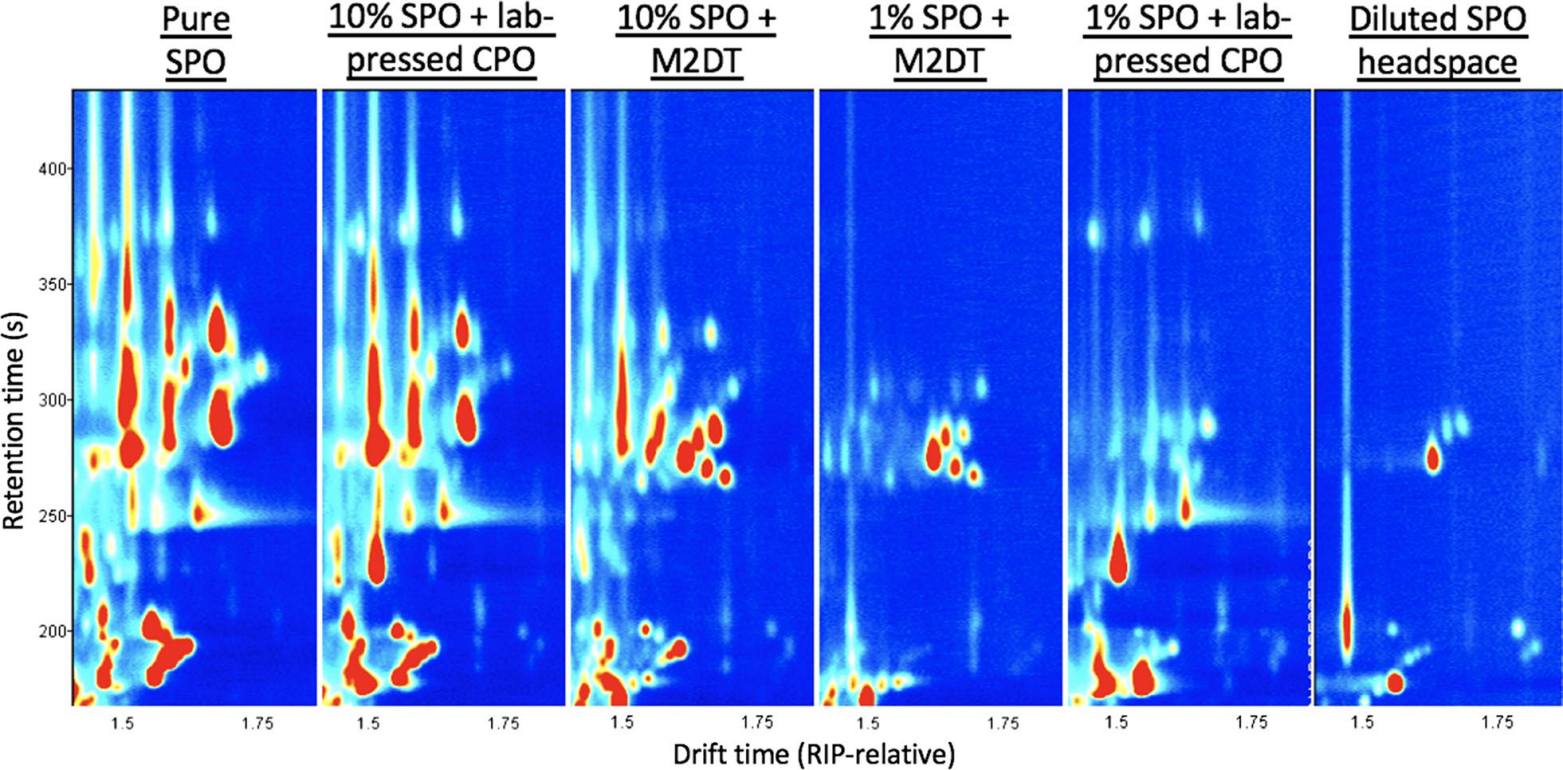
Reporter view showing region 4 of the SPO fingerprint across a range of samples. 10% SPO + lab-pressed CPO and 10% SPO + Mill 2 Dispatch Tank (M2DT) are most similar to the pure SPO fingerprint. 1% SPO + M2DT CPO and 1% SPO + lab-pressed CPO share similarities with both pure SPO and diluted SPO headspace. This trend is repeated for all regions of the SPO fingerprint across all spiked lab-pressed CPO and DT CPO (data not shown)

DT control samples were compared to SPO-spiked DT samples and a significant SPO fingerprint was always present in the spiked samples. None of the DT control samples contained any SPO-related peaks, indicating they were free of SPO contamination. The fingerprint of the SPO-spiked DT samples was compared to that of pure SPO and the diluted headspace of SPO, to determine whether it was similar. For SPO-spiked DT samples containing 5% SPO or greater, the SPO fingerprint was similar to that of pure SPO and of the lab-pressed CPO spiked with 10% SPO. When SPO-spiked DT samples contained less that 5% SPO, the SPO fingerprint was similar to that of the diluted SPO headspace and of the lab-pressed CPO spiked with 1% SPO. The 21 previously determined SPO markers matched with those found in SPO-spiked DT samples (Fig. 3). The influence of geographical origin, processing differences etc., did not appear to interfere with the SPO fingerprint.

To our knowledge, there is only one other study which has sought to detect SPO contamination in CPO and none for the detection of PFO in CPO. The aforementioned SPO study compared the dielectric constants of CPO to those of CPO spiked with SPO (0.6%, 1.0%, 5.0% and 10.0%) and relied upon the moisture content of SPO. Like this present study, it was also able to detect SPO at low concentrations [18]. However, this present study suggests a reliable SPO VOC fingerprint which can be rapidly screened for on-site, down to a 1% concentration, with the potential to identify specific VOCs pending further work.

### Conclusions

We believe that GC–IMS is a suitable technique for initial screening of CPO to detect the presence of unwanted residual oils. Detection of a reliable PFO fingerprint in spiked DT samples was not possible, potentially due to DT control samples already containing PFO. However, a significant SPO fingerprint was detected in all spiked DT samples and spiked lab-pressed CPO (down to 1% (w/w) SPO). The SPO fingerprint was characterised by 21 VOCs and spanned four spectral regions.

## Limitations

The study should also be replicated using a larger sample size and a range of SPO and PFO samples from different sources to ensure the fingerprint is consistent and variation according to geography/processing etc. does not impact the proposed fingerprint. Further work should now be done to determine the limit of detection (LOD) and limit of quantification (LOQ) of SPO and to discover the chemical identities of the marker VOCs.

## Additional files

**Additional file 1: Table S1.** List of crude palm oil samples provided for the study.

**Additional file 2: Table S2.** The instrumental and experimental parameters for the study.

**Additional file 3: Table S3.** Measured intra- and interday precision values for each working standard solution.

**Additional file 4: Figure S1.** Side-by-side comparison of lab-pressed CPO, pure PFO and diluted headspace of SPO spectra.

**Additional file 5: Table S4.** Possible PFO markers determined by spiking lab-pressed CPO with pure PFO.

**Additional file 6: Table S5.** Possible SPO markers determined by spiking lab-pressed CPO with pure SPO.

**Additional file 7: Figure S2.** A full view of the SPO fingerprint and its individual markers.

## Abbreviations

CPO: crude palm oil
DT: dispatch tank
GC–IMS: gas chromatography–ion mobility spectrometry
GC–MS: gas chromatography–mass spectrometry
HS-SPME: headspace solid-phase microextraction
LAV: Laboratory Analytical Viewer
LOD: limit of detection
LOQ: limit of quantification
MCPDs: monochloropropanediols
PFO: palm fibre oil
PTR-MS: proton transfer reaction-mass spectrometry
SPO: sludge palm oil
VOC: volatile organic compounds
