# Supplementary material for: Use of headspace–gas chromatography–ion mobility spectrometry to detect volatile fingerprints of palm fibre oil and sludge palm oil in samples of crude palm oil
Source: BMC Res Notes. 2019 Apr 16;12:229. doi: 10.1186/s13104-019-4263-7 (PMC6469128; doi:10.1186/s13104-019-4263-7)
Supplement: Supplementary file 1 — Additional file 1: Table S1. List of crude palm oil samples provided for the study. [file 13104_2019_4263_MOESM1_ESM.docx]

**Table S1. List of crude palm oil samples provided for the study**

| **Mill** | **Sample type** |
| --- | --- |
| 1 | Dispatch tank (DT) CPO (control) |
| 1 | DT CPO + 1% PFO |
| 1 | DT CPO + 2% PFO |
| 1 | DT CPO + 4% PFO |
| 1 | DT CPO + 6% PFO |
| 1 | DT CPO + 1% SPO |
| 1 | DT CPO + 2.5% SPO |
| 1 | DT CPO + 5% SPO |
| 1 | DT CPO + 7.5% SPO |
| 1 | DT CPO + 10% SPO |
| 2 | DT CPO (control) |
| 2 | DT CPO + 1% PFO |
| 2 | DT CPO + 2% PFO |
| 2 | DT CPO + 4% PFO |
| 2 | DT CPO + 6% PFO |
| 2 | DT CPO + 1% SPO |
| 2 | DT CPO + 2.5% SPO |
| 2 | DT CPO + 5% SPO |
| 2 | DT CPO + 7.5% SPO |
| 2 | DT CPO + 10% SPO |
| 3 | DT CPO (control) |
| 3 | DT CPO + 1% PFO |
| 3 | DT CPO + 2% PFO |
| 3 | DT CPO + 4% PFO |
| 3 | DT CPO + 6% PFO |
| 3 | DT CPO + 1% SPO |
| 3 | DT CPO + 2.5% SPO |
| 3 | DT CPO + 5% SPO |
| 3 | DT CPO + 7.5% SPO |
| 3 | DT CPO + 10% SPO |
| 4 | DT CPO (control) |
| 4 | DT CPO + 1% PFO |
| 4 | DT CPO + 2% PFO |
| 4 | DT CPO + 4% PFO |
| 4 | DT CPO + 6% PFO |
| 4 | DT CPO + 1% SPO |
| 4 | DT CPO + 2.5% SPO |
| 4 | DT CPO + 5% SPO |
| 4 | DT CPO + 7.5% SPO |
| 4 | DT CPO + 10% SPO |
