# Supplementary material for: Use of headspace–gas chromatography–ion mobility spectrometry to detect volatile fingerprints of palm fibre oil and sludge palm oil in samples of crude palm oil
Source: BMC Res Notes. 2019 Apr 16;12:229. doi: 10.1186/s13104-019-4263-7 (PMC6469128; doi:10.1186/s13104-019-4263-7)
Supplement: Supplementary file 2 — Additional file 2: Table S2. The instrumental and experimental parameters for the study. [file 13104_2019_4263_MOESM2_ESM.docx]

**Table S2. The instrumental and experimental parameters for the study**

| Parameter | Values and units |
| --- | --- |
| **Sample Introduction System** |  |
| Sampling type/volume | Headspace (200 $\mu$L) |
| Agitation time | 15 min |
| Sample volume | 1 mL |
| Incubation temperature | 60 ^o^C |
| Injector temperature | 80 ^o^C |
| Syringe temperature | 80 ^o^C |
| **Column** |  |
| Capillary Column | SE-54 (low polar) ID 0.53mm, 1μm |
| Column Length | 15 m |
| Column Temperature | 40 ^o^C |
| Run time | 21 min |
| Carrier gas flow rate | Ramped flow programme |
| **IMS** |  |
| Ionization source | Tritium (30 MBq) |
| Voltage | Positive drift |
| Drift length | 9.8 cm |
| Electric field strength | 510 V cm^-1^ |
| Drift gas flow rate | 150 mL min^-1^ |
| IMS temperature | 45 ^o^C |
