# Supplementary material for: Use of headspace–gas chromatography–ion mobility spectrometry to detect volatile fingerprints of palm fibre oil and sludge palm oil in samples of crude palm oil
Source: BMC Res Notes. 2019 Apr 16;12:229. doi: 10.1186/s13104-019-4263-7 (PMC6469128; doi:10.1186/s13104-019-4263-7)
Supplement: Supplementary file 3 — Additional file 3: Table S3. Measured intra- and interday precision values for each working standard solution. [file 13104_2019_4263_MOESM3_ESM.docx]

**Table S3. Measured intra- and interday precision values for each working standard solution.**

|  | | **Repeatability**  **(n=5)** | **Intermediate precision**  **(n=7)** |
| --- | --- | --- | --- |
| **2-butanone** | **TR** | 0.45% | 0.41% |
|  | **TD** | 0.29% | 0.31% |
|  | **I** | 1.14% | 1.69% |
| **2-hexanone** | **TR** | 0.35% | 0.85% |
|  | **TD** | 0.05% | 0.29% |
|  | **I** | 0.50% | 0.64% |
| **2-nonanone** | **TR** | 0.20% | 0.72% |
|  | **TD** | 0.03% | 0.31% |
|  | **I** | 0.56% | 1.13% |

*TR: retention time, TD: drift time, I: peak intensity
