# Supplementary material for: Use of headspace–gas chromatography–ion mobility spectrometry to detect volatile fingerprints of palm fibre oil and sludge palm oil in samples of crude palm oil
Source: BMC Res Notes. 2019 Apr 16;12:229. doi: 10.1186/s13104-019-4263-7 (PMC6469128; doi:10.1186/s13104-019-4263-7)
Supplement: Supplementary file 4 — Additional file 4: Figure S1. Side-by-side comparison of lab-pressed CPO, pure PFO and diluted headspace of SPO spectra. [file 13104_2019_4263_MOESM4_ESM.docx]

**Figure S1. Side-by-side comparison of lab-pressed CPO, pure PFO and diluted headspace of SPO spectra.**


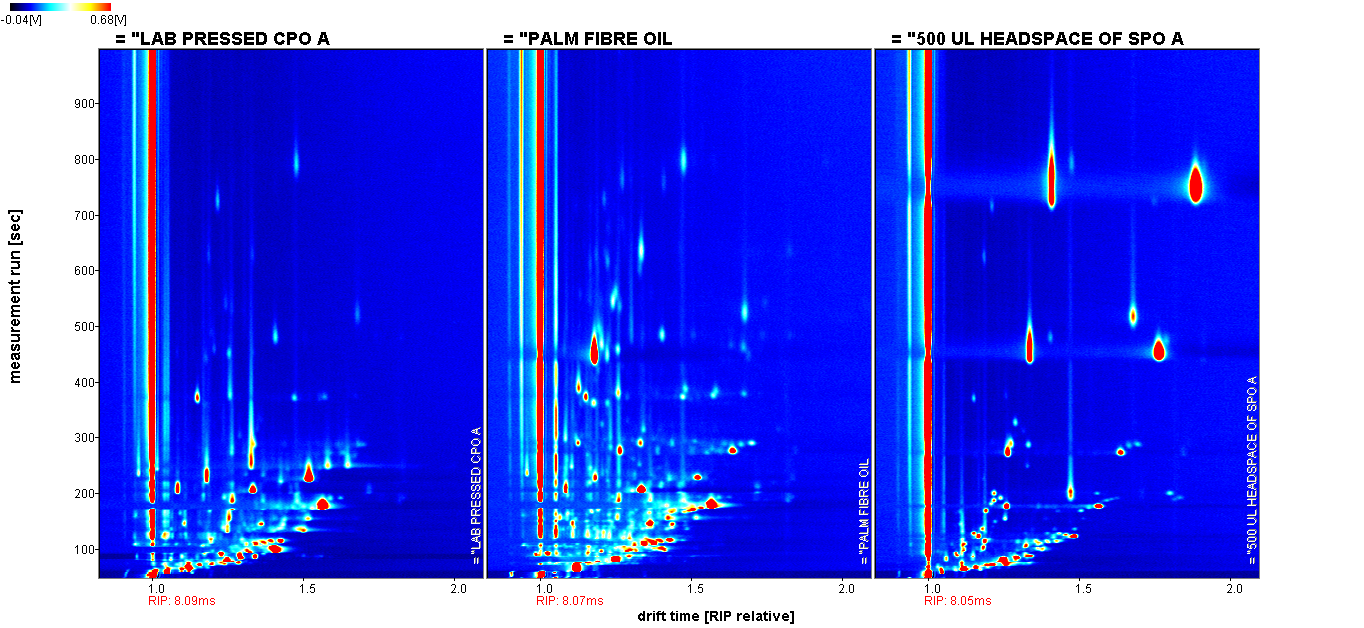

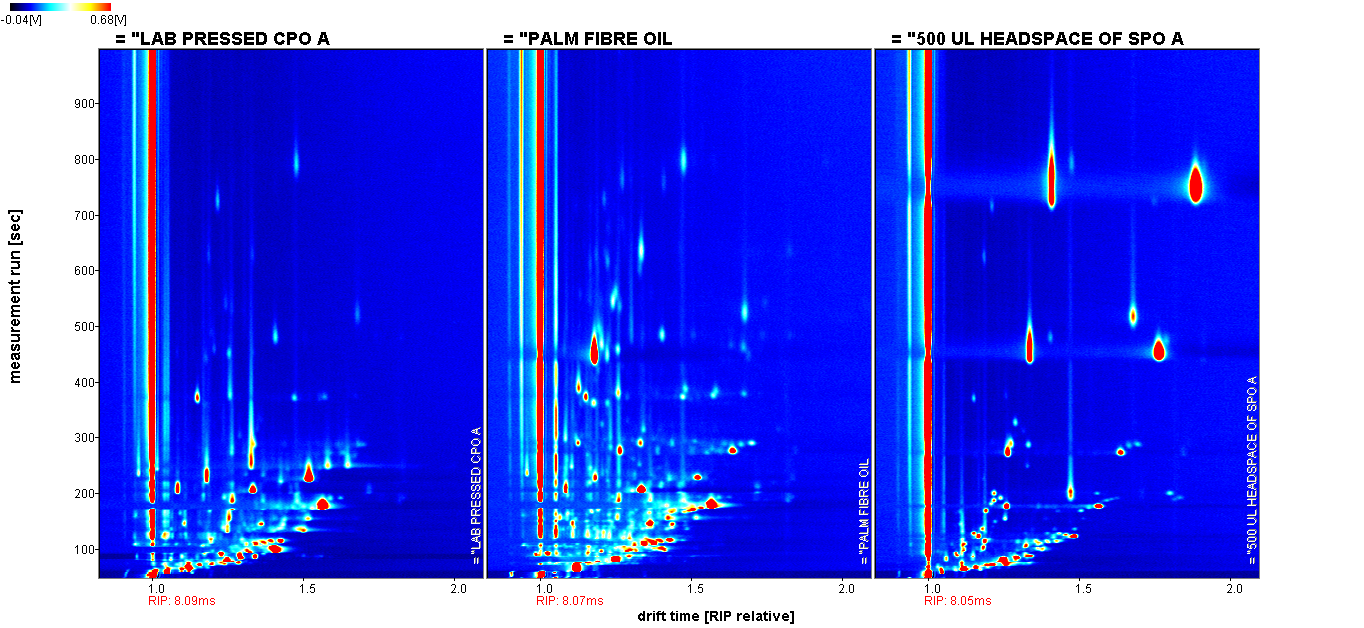

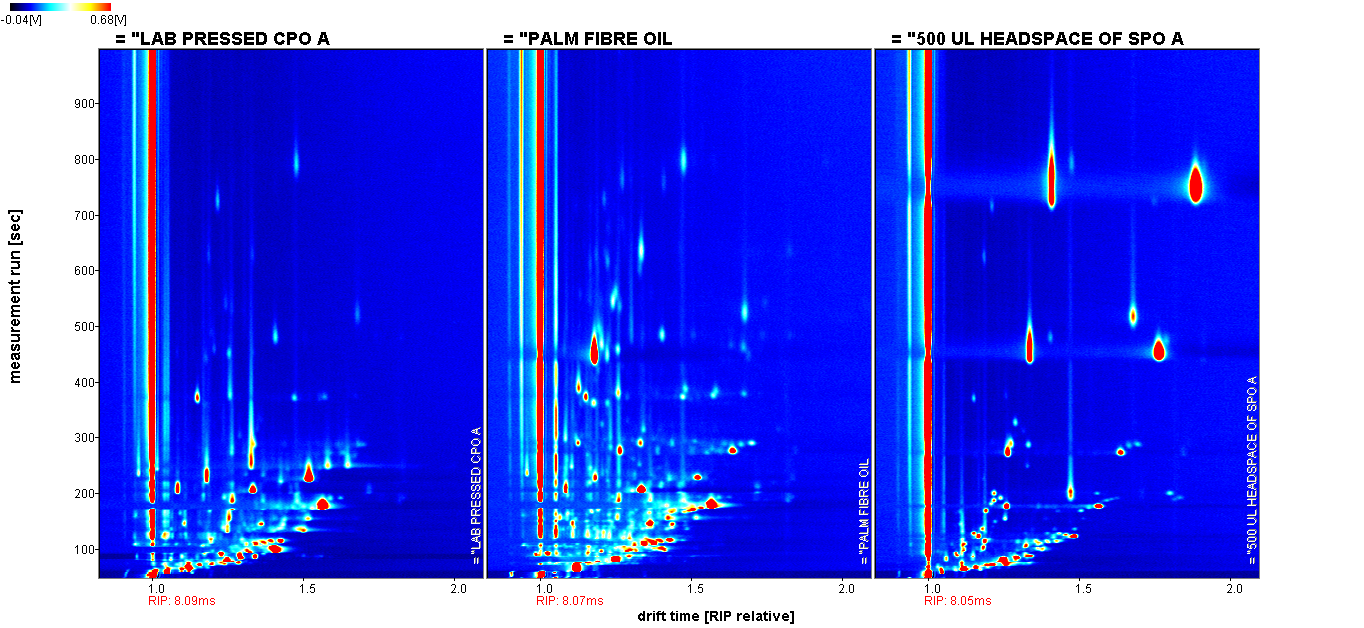


Lab-pressed CPO Palm Fibre oil Sludge Palm oil
