# Supplementary material for: Use of headspace–gas chromatography–ion mobility spectrometry to detect volatile fingerprints of palm fibre oil and sludge palm oil in samples of crude palm oil
Source: BMC Res Notes. 2019 Apr 16;12:229. doi: 10.1186/s13104-019-4263-7 (PMC6469128; doi:10.1186/s13104-019-4263-7)
Supplement: Supplementary file 5 — Additional file 5: Table S4. Possible PFO markers determined by spiking lab-pressed CPO with pure PFO. [file 13104_2019_4263_MOESM5_ESM.docx]

**Table S4. Possible PFO markers determined by spiking lab-pressed CPO with pure PFO**

| **Markers** | **Retention Index** | **Drift time (ms) (RIP Rel)** | **1/K_0_**  (cm ^2^ s^-1^ V^-1^) |
| --- | --- | --- | --- |
| M1 | 450.961 | 1.1787 | 0.58 |
| M2 | 390.700 | 1.1271 | 0.55 |
| M3 | 903.900 | 1.1255 | 0.55 |
| M4 | 893.300 | 1.6369 | 0.80 |
| M5 | 950.200 | 1.1778 | 0.58 |
| M6 | 786.700 | 1.5069 | 0.74 |
| M7 | 380.373 | 1.6760 | 0.82 |
