# Supplementary material for: Use of headspace–gas chromatography–ion mobility spectrometry to detect volatile fingerprints of palm fibre oil and sludge palm oil in samples of crude palm oil
Source: BMC Res Notes. 2019 Apr 16;12:229. doi: 10.1186/s13104-019-4263-7 (PMC6469128; doi:10.1186/s13104-019-4263-7)
Supplement: Supplementary file 6 — Additional file 6: Table S5. Possible SPO markers determined by spiking lab-pressed CPO with pure SPO. [file 13104_2019_4263_MOESM6_ESM.docx]

**Table S5. Possible SPO markers determined by spiking lab-pressed CPO with pure SPO**

| **Markers** | **Fingerprint Region** | **Retention Index** | **Drift time (ms) (RIP Rel)** | **1/K_0_**  (cm ^2^ s^-1^ V^-1^) |
| --- | --- | --- | --- | --- |
| M1 | 1 | 1103.8 | 1.4066 | 0.69 |
| M2 | 2 | 1008.9 | 1.3056 | 0.64 |
| M3 | 2 | 1002.6 | 1.6432 | 0.81 |
| M4 | 2 | 1000.1 | 1.6970 | 0.83 |
| M5 | 2 | 1006.3 | 1.7147 | 0.84 |
| M6 | 2 | 1007.2 | 1.8090 | 0.89 |
| M7 | 3 | 897.20 | 1.6593 | 0.81 |
| M8 | 3 | 900.30 | 1.6915 | 0.83 |
| M9 | 3 | 893.30 | 1.5684 | 0.77 |
| M10 | 3 | 899.50 | 1.5831 | 0.78 |
| M11 | 3 | 912.60 | 1.6040 | 0.79 |
| M12 | 3 | 928.80 | 1.6838 | 0.83 |
| M13 | 4 | 900.90 | 1.5127 | 0.74 |
| M14 | 4 | 811.70 | 1.6234 | 0.80 |
| M15 | 4 | 818.10 | 1.5835 | 0.78 |
| M16 | 4 | 821.20 | 1.5578 | 0.76 |
| M17 | 4 | 811.90 | 1.4893 | 0.73 |
| M18 | 4 | 784.10 | 1.5078 | 0.74 |
| M19 | 4 | 794.60 | 1.4846 | 0.73 |
| M20 | 4 | 781.90 | 1.3593 | 0.67 |
| M21 | 4 | 700.40 | 1.3727 | 0.67 |

* RIP Rel: meaning the RIP (Reactant Ion Peak) of each file has been aligned to one another.
