# Supplementary material for: Use of headspace–gas chromatography–ion mobility spectrometry to detect volatile fingerprints of palm fibre oil and sludge palm oil in samples of crude palm oil
Source: BMC Res Notes. 2019 Apr 16;12:229. doi: 10.1186/s13104-019-4263-7 (PMC6469128; doi:10.1186/s13104-019-4263-7)
Supplement: Supplementary file 7 — Additional file 7: Figure S2. A full view of the SPO fingerprint and its individual markers. [file 13104_2019_4263_MOESM7_ESM.docx]

**Figure S2. A full view of the SPO fingerprint and its individual markers.** An expanded view of regions 1 to 4 is shown in the three panels on the right. Marker numbers 1-21 correspond with the information in Table 5.

**
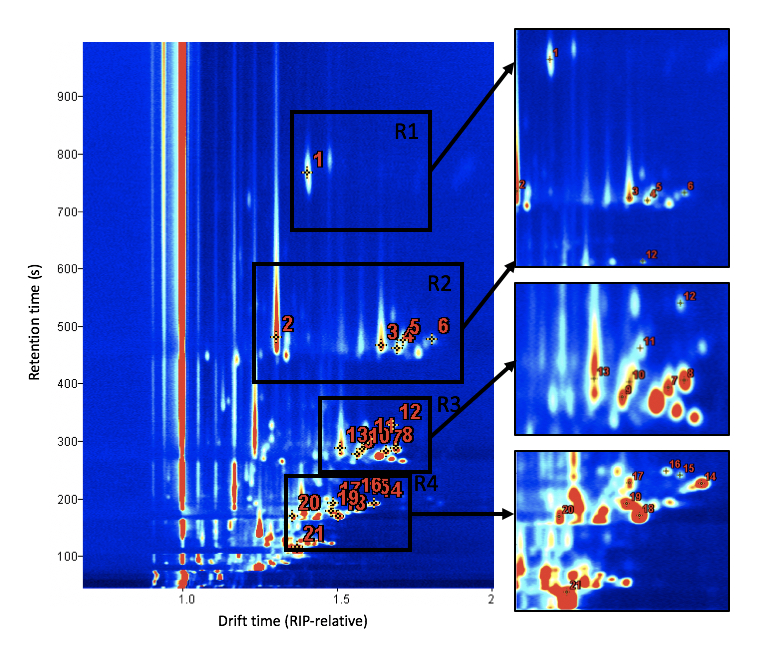
**
